# Lysosomal Ca^2+^ flux modulates automaticity in ventricular cardiomyocytes and correlates with arrhythmic risk

**DOI:** 10.1093/pnasnexus/pgad174

**Published:** 2023-05-25

**Authors:** An Xie, Gyeoung-Jin Kang, Eun Ji Kim, Feng Feng, Sophie E Givens, Brenda M Ogle, Samuel C Dudley

**Affiliations:** Department of Medicine, University of Minnesota, 401 East River Parkway, VCRC 1st Floor, Suite 131, Minneapolis, MN 55455, USA; Lillehei Heart Institute, University of Minnesota, 2231 6th Street SE, Suite 4-156, Minneapolis, MN 55455, USA; Department of Medicine, University of Minnesota, 401 East River Parkway, VCRC 1st Floor, Suite 131, Minneapolis, MN 55455, USA; Lillehei Heart Institute, University of Minnesota, 2231 6th Street SE, Suite 4-156, Minneapolis, MN 55455, USA; Department of Medicine, University of Minnesota, 401 East River Parkway, VCRC 1st Floor, Suite 131, Minneapolis, MN 55455, USA; Lillehei Heart Institute, University of Minnesota, 2231 6th Street SE, Suite 4-156, Minneapolis, MN 55455, USA; Department of Medicine, University of Minnesota, 401 East River Parkway, VCRC 1st Floor, Suite 131, Minneapolis, MN 55455, USA; Lillehei Heart Institute, University of Minnesota, 2231 6th Street SE, Suite 4-156, Minneapolis, MN 55455, USA; Department of Biomedical Engineering, Stem Cell Institute, University of Minnesota, McGuire Translational Research Facility, 2001 6th Street SE, Mail Code 2873, Minneapolis, MN 55455, USA; Lillehei Heart Institute, University of Minnesota, 2231 6th Street SE, Suite 4-156, Minneapolis, MN 55455, USA; Department of Biomedical Engineering, Stem Cell Institute, University of Minnesota, McGuire Translational Research Facility, 2001 6th Street SE, Mail Code 2873, Minneapolis, MN 55455, USA; Department of Pediatrics, Institute for Engineering in Medicine, University of Minnesota, 420 Delaware Street Southeast, 725 Mayo Memorial Building, MMC 94, Minneapolis, MN 55455, USA; Department of Medicine, University of Minnesota, 401 East River Parkway, VCRC 1st Floor, Suite 131, Minneapolis, MN 55455, USA; Lillehei Heart Institute, University of Minnesota, 2231 6th Street SE, Suite 4-156, Minneapolis, MN 55455, USA

**Keywords:** automaticity, lysosome, calcium, human-induced pluripotent stem cell, cardiomyocytes

## Abstract

Automaticity involves Ca^2+^ handling at the cell membrane and sarcoplasmic reticulum (SR). Abnormal or acquired automaticity is thought to initiate ventricular arrhythmias associated with myocardial ischemia. Ca^2+^ flux from mitochondria can influence automaticity, and lysosomes also release Ca^2+^. Therefore, we tested whether lysosomal Ca^2+^ flux could influence automaticity. We studied ventricular human-induced pluripotent stem cell–derived cardiomyocytes (hiPSC-CMs), hiPSC 3D engineered heart tissues (EHTs), and ventricular cardiomyocytes isolated from infarcted mice. Preventing lysosomal Ca^2+^ cycling reduced automaticity in hiPSC-CMs. Consistent with a lysosomal role in automaticity, activating the transient receptor potential mucolipin channel (TRPML1) enhanced automaticity, and two channel antagonists reduced spontaneous activity. Activation or inhibition of lysosomal transcription factor EB (TFEB) increased or decreased total lysosomes and automaticity, respectively. In adult ischemic cardiomyocytes and hiPSC 3D EHTs, reducing lysosomal Ca^2+^ release also inhibited automaticity. Finally, TRPML1 was up-regulated in cardiomyopathic patients with ventricular tachycardia (VT) compared with those without VT. In summary, lysosomal Ca^2+^ handling modulates abnormal automaticity, and reducing lysosomal Ca^2+^ release may be a clinical strategy for preventing ventricular arrhythmias.

**Significance Statement**

Abnormal ventricular automaticity occurs in injured myocardium and is thought to be one mechanism of enhanced arrhythmic risk. Enhanced and reduced lysosomal Ca^2+^ handling accelerated or slowed automaticity in ventricular cells derived from human-induced pluripotent stem cells or cardiomyocytes isolated from infarcted hearts. A lysosomal Ca^2+^ release channel was correlated with arrhythmic risk in humans, and inhibition of lysosomal Ca^2+^ release prevented arrhythmias in isolated hearts, suggesting that lysosomal Ca^2+^ release may contribute to arrhythmic risk.

## Introduction

Acquired or abnormal automaticity is thought to contribute to arrhythmias and sudden death in ischemic heart disease (1), and the mechanisms of abnormal automaticity in usually quiescent cardiomyocytes (CMs) are thought to be similar to those in pacemaker cells. The mechanisms of pacemaker automaticity are described in terms of two Ca^2+^ cycling systems, the membrane and the intracellular Ca^2+^ clocks (2, 3). These determinants of automaticity are modulated by release of Ca^2+^ from the sarcoplasmic reticulum (SR) through the ryanodine receptor type 2 (RyR2). SR Ca^2+^ release has its effect by increasing a depolarizing current mediated by the Na^+^/Ca^2+^ exchanger (NCX) (4–7). Other ion channels and receptors demonstrated to be involved in automaticity in various types of CMs include the inward rectifier potassium current (I_K1_) (8), the I_f_ pacemaker current (9), and the SR inositol trisphosphate receptor (IP_3_R) (10).

Since Ca^2+^ cycling is central to automaticity, we tested whether mitochondrial Ca^2+^ cycling could play a role in automaticity. We demonstrated a role for mitochondrial Ca^2+^ cycling in abnormal automaticity in ventricular CMs, including mouse embryonic stem cell (ESC)–derived ventricular-like CMs, human-induced pluripotent stem cell (hiPSC)–derived ventricular-like CMs (hiPSC-CMs), and ischemic adult mouse ventricular CMs (11). In addition, mitochondrial Ca^2+^ cycling has been shown to modulate cardiac pacemaker and atrial cell automaticity (12, 13). An important element of mitochondrial Ca^2+^ cycling includes Ca^2+^ uptake through the mitochondrial Ca^2+^ uniporter (MCU) (14). Mitochondrial Ca^2+^ efflux occurs mainly through the mitochondrial NCX (15).

Recently, lysosome-cardiac SR contacts have been reported (16). SR Ca^2+^ release may contribute to lysosomal Ca^2+^ loading (17), and lysosomal Ca^2+^ release can trigger SR Ca^2+^ release in noncardiac tissues (18–20). Based upon these publications, it is reasonable to test if lysosomal Ca^2+^ flux may influence automaticity via Ca^2+^ release in CMs.

Like mitochondria, lysosomes accumulate and release Ca^2+^. Accumulation of Ca^2+^ occurs in a coupled process where protons are pumped into lysosomes by the V-type H^+^-ATPase (V-ATPase) and protons are exchanged for Ca^2+^ by an H^+^/Ca^2+^ exchanger in CMs (21). Therefore, Ca^2+^ concentration in lysosomes is significantly higher than that in the cytosol (∼500 µmol/L in lysosomes) (21, 22). Lysosome Ca^2+^ release is mediated by a type 2 two-pore channel (TPC2) and a transient receptor potential mucolipin channel (TRPML1) (21). The Ca^2+^-permeable, nonselective cation channel known as TRPML1 is found exclusively on the membranes of late-stage endosomes and lysosomes, and it is not present or active on the plasma membrane (18, 23). Lysosomal number and TRPML1 transcription is dependent on transcription factor EB (TFEB), a master regulator of the autophagy-lysosome pathway, which can be modified or activated by reactive oxygen species (ROS) (24).

The spontaneous electrical activity of hiPSC-CMs provides an opportunity for a more in-depth examination of the ion currents that drive ventricular automaticity (25). Therefore, hiPSC-CMs, together with adult ischemic mouse ventricular cells, were used to test whether lysosomal Ca^2+^ flux can modulate ventricular automaticity.

## Results

### Lysosomal Ca^2+^ flux–modulated automaticity

**Fig. 1.**
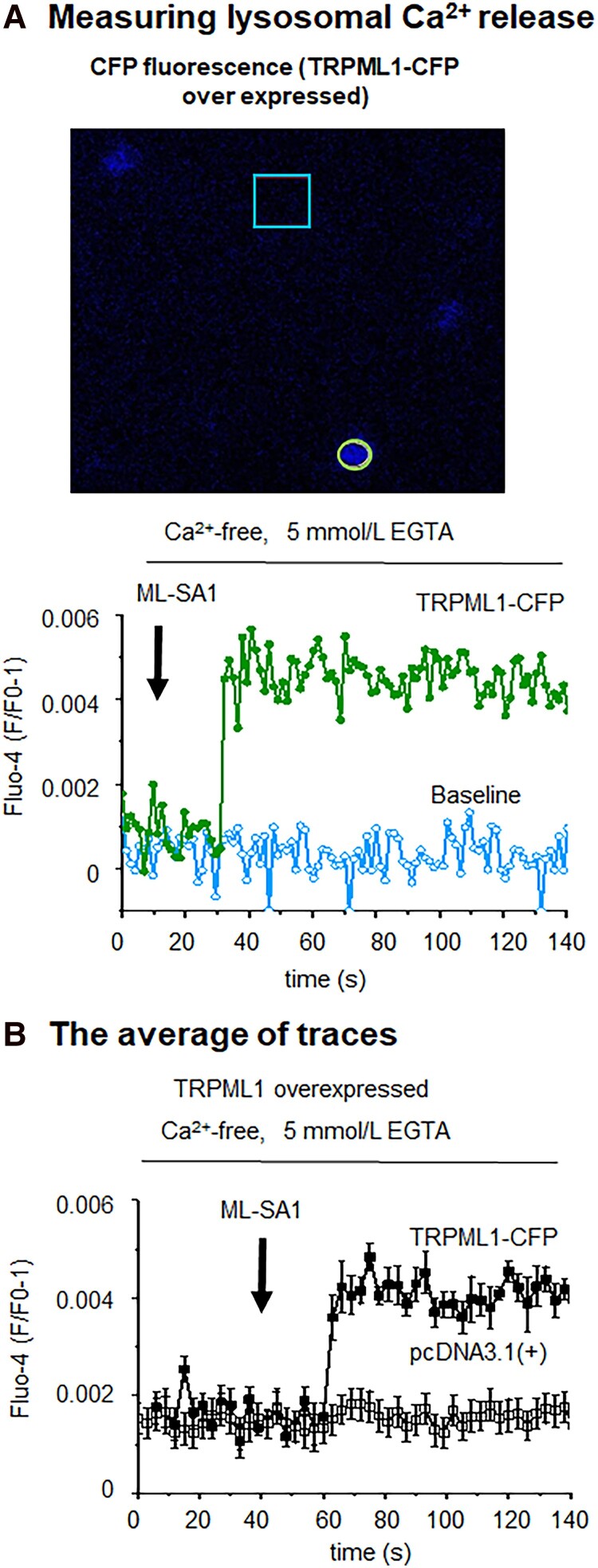
Lysosomal Ca^2+^ release in hiPSC-CMs overexpressing TRPML1. Cells were loaded with Fluo-4 for imaging. The TRPML1-specific agonist, ML-SA1 500 nmol/L, could induce lysosomal Ca^2+^ release in a Ca^2+^ free bath solution from TRPML1-CFP overexpressing hiPSC-CMs while ML-SA1 could not induce lysosomal Ca^2+^ release in pcDNA3.1(+) control cells. A) Raw data in TRPML1-CFP overexpressing hiPSC-CMs. CFP acted as a transfection marker. Fluo-4 fluorescence for TRPML1 Ca^2+^ release was measured from the area marked by the green ellipse. Background was defined as the light blue rectangular box. B) Lysosomal Ca^2+^ traces represent the average of 10 and 14 cells in TRPML1 overexpressed (solid cycle) and their pcDNA3.1(+) control (open cycle) hiPSC-CMs, respectively. Data are represented as mean ± SEM.

**Fig. 2.**
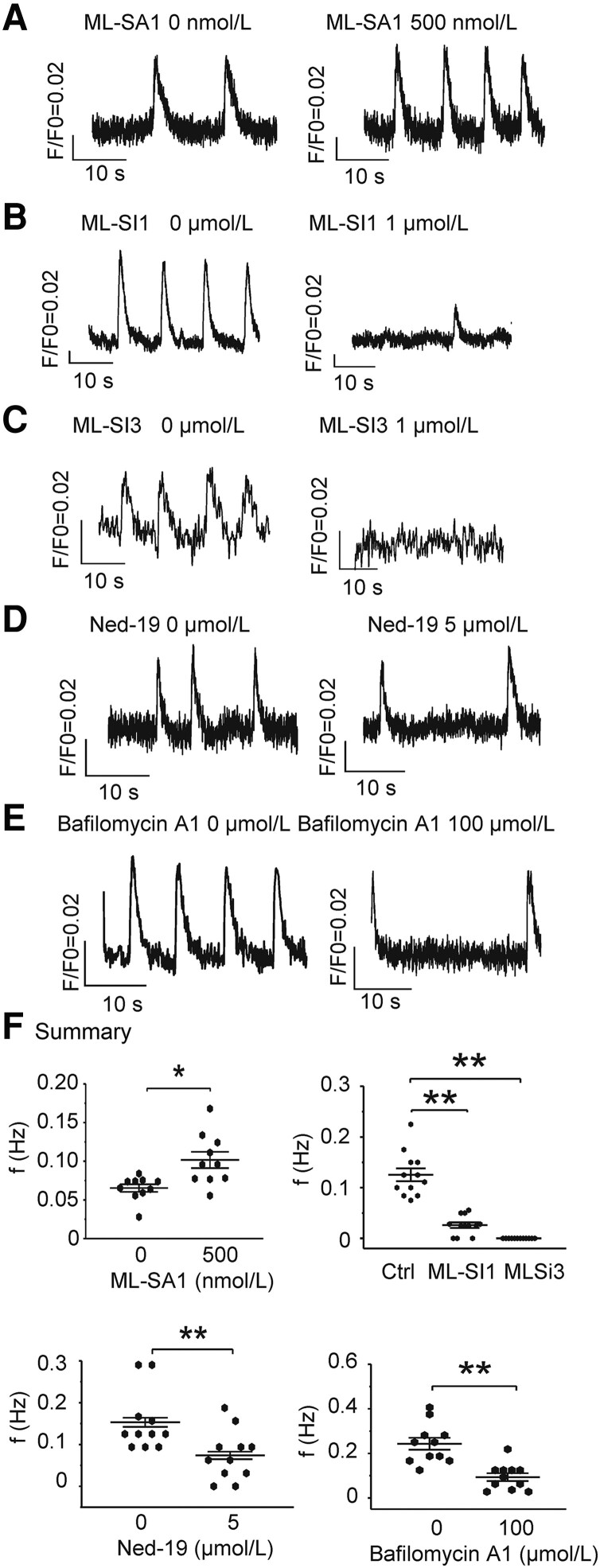
Lysosomal Ca^2+^ flux modulates the automaticity of hiPSC-CMs. A) The TRPML1-specific agonist, ML-SA1 500 nmol/L, could up-regulate beating frequency. B) The TRPML1-specific antagonist, ML-SI1 1 µmol/L, could down-regulate spontaneous beating rate. C) A second TRPML1-specific antagonist, ML-S31 1 µmol/L, could abolish spontaneous beating. D) The TPC2 specific blocker (Ned-19, 5 µmol/L) could decrease automaticity of hiPSC-CMs. E) The V-type H^+^ ATPase specific inhibitor (bafilomycin A1, 100 µmol/L) could reduce the hiPSC-CM beating rate. F) The summary. *n* = 10, 12, 12, 11, and 11 in A, B, C, D, and E, respectively. Data are represented as mean ± SEM. *P < 0.05, compared between two indicated groups by paired *t*-test. The two traces in each panel (A–E) are from the same cell.

Since Ca^2+^ cycling is central to automaticity and TRPML1 is considered to be the principal Ca^2+^ release channel in the lysosome (26), the effect of alteration of TRPML1 on the automaticity was tested. The TRPML1-specific agonist (22), ML-SA1 (500 nmol/L), could induce lysosome Ca^2+^ release from hiPSC-CMs overexpressing TRPML1 in a Ca^2+^-free bath solution (Fig. 1). As shown in Fig. 2A, ML-SA1 (500 nmol/L) could significantly increase the beating rate (*P <* 0.05), while the TRPML1-specific antagonists (22), ML-SI1 or ML-SI3 (1 µmol/L), could reduce this rate (Fig. 2B and C, *P <* 0.01). The effect of blocking another lysosome Ca^2+^ release channel (TPC2) by 5 µmol/L Ned-19 on beating rate was similar to that of inhibition of TRPML1 (Fig. 2D, *P <* 0.01). Inhibiting the V-ATPase prevents proton flux into lysosomes, reduces the H^+^/Ca^2+^ exchanger activity, and depletes lysosomal Ca^2+^ stores (27). The V-ATPase specific inhibitor, bafilomycin A1 (100 µmol/L), decreased spontaneous beating (Fig. 2E, *P <* 0.01), supporting the role of lysosomes in automaticity. As expected for a small Ca^2+^ release, pharmacological manipulation of lysosomal Ca^2+^ release did not influence basal Ca^2+^ or cellular Ca^2+^ transients, except ML-SI1 which reduced the amplitude of the Ca^2+^ transients by 41% for unclear reasons (Fig. 2).

### Silencing lysosomal TRPML1 channels could reduce automaticity

**Fig. 3.**
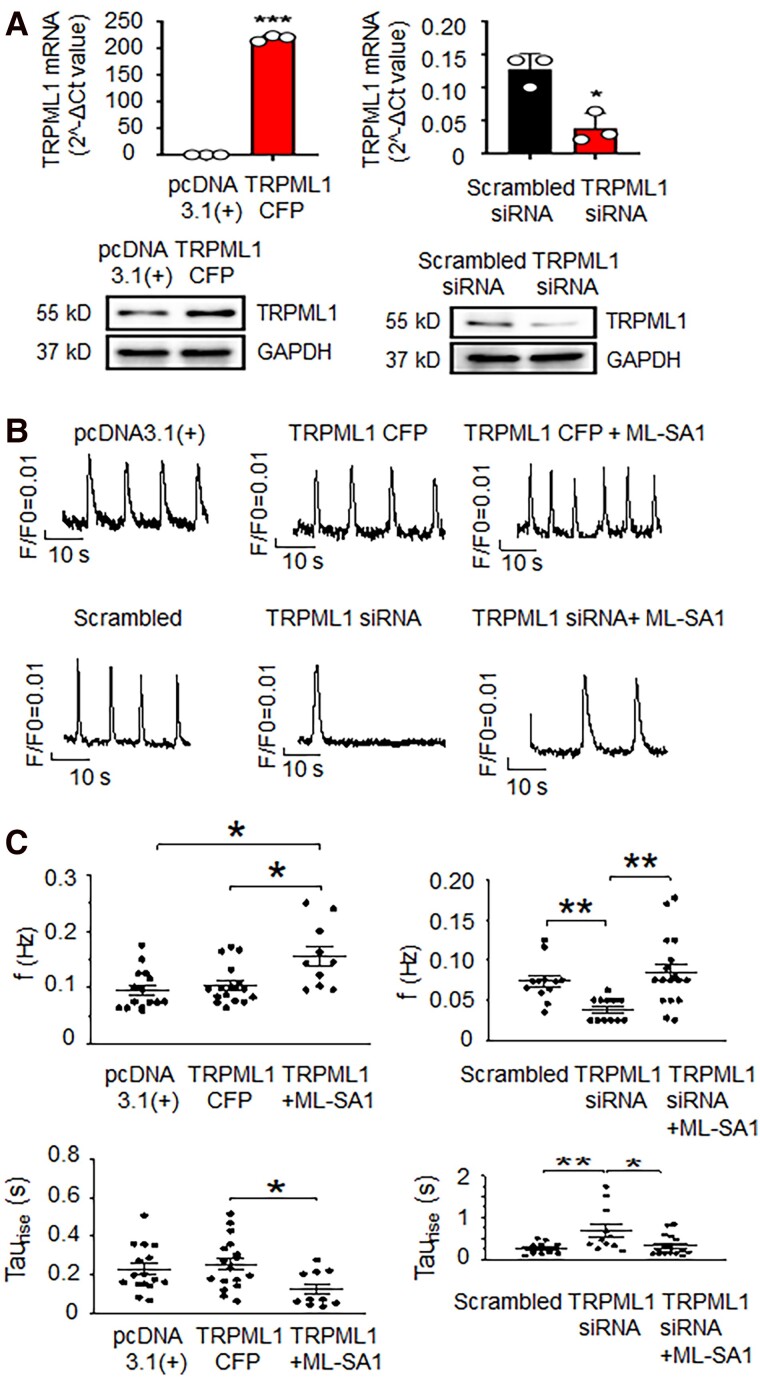
Down-regulation of lysosomal TRPML1 channels in hiPSC-CMs could reduce spontaneous beating. A) The mRNA level of TRPML1 was determined by qPCR. Control (pcDNA3.1(+) or scrambled siRNA), TRPML1-CFP, and TRPML1 siRNA were transfected into hiPSC-CMs. Data are represented as mean ± SEM. *P < 0.05 and **P < 0.01, compared with that in control group by the Mann–Whitney test. *n* = 3 for each group. The protein level of TRPML1 was determined by Western blot. B) Typical Ca^2+^ transients recorded from control, TRPML1 overexpression, TRPML1 overexpression with ML-SA1 500 nmol/L, scrambled siRNA, TRPML1 siRNA, and TRPML1 siRNA with ML-SA1 500 nmol/L groups. C) The statistical analysis of spontaneous beating frequency and Ca^2+^ transient rise time constants from the data in B). Data are represented as mean ± SEM. *P < 0.05, **P < 0.01 compared with that between two indicated groups by one-way ANOVA testing with Bonferroni correction for multiple comparisons was used. *n* = 16, 17, 10, 12, 12, and 17 for pcDNA3.1(+) control, TRPML1 overexpression, TRPML1 overexpression with ML-SA1 500 nmol/L, scrambled control, TRPML1 siRNA, and TRPML1 siRNA with ML-SA1 500 nmol/L groups. respectively.

Pharmacological tools are subject to possible off-target effects, so we tested the effect of TRPML1 modulation by genetic means. As shown in Fig. 3A, the mRNA expression of TRPML1 was significantly increased after TRPML1-cyan fluorescent protein (CFP) was transfected into hiPSC-CMs (*P <* 0.01). On the other hand, the mRNA expression of TRPML1 was significantly decreased after TRPML1 siRNA transfection (*P <* 0.05, Fig. 3A). The alteration of TRPML1 protein levels was consistent with the changes in mRNA. Down-regulation of TRPML1 could slow greatly the intracellular Ca^2+^ transient rise phase and reduce the automaticity of hiPSC-CMs (Fig. 3B and C, *P <* 0.05). Up-regulation of TRPML1 did not substantially accelerate SR Ca^2+^ release or the spontaneous beating in hiPSC-CMs, presumably because TRPML1 overexpression alone did not increase lysosomal Ca^2+^ release (Fig. 3B and C, *P >* 0.05). Despite this, increasing TRPML1 channel activity by the TRPML1 agonist ML-SA1 could increase automaticity, while TRPML1 inhibition reduced automaticity (Fig. 3B and C). Two-way ANOVA tests using the independent variables of treatment and cell batch showed no significant differences among cell batches.

### Modification of TRPML1 expression by TFEB nuclear translocation could affect spontaneous beating in hiPSC-CMs

Lysosome number and TRPML1 transcription are dependent on TFEB thought to be the most important transcription factor regulating the autophagy-lysosome pathway (24). Lysosomal Ca^2+^ release through TRPML1 channels is responsible for TFEB dephosphorylation and translocation to the nucleus, where the transcription factor causes increased lysosomal biogenesis and TRPML1 transcription (28). This creates a positive feedback loop whereby increased TRPML1 activity increases TRPML1 transcription.

**Fig. 4.**
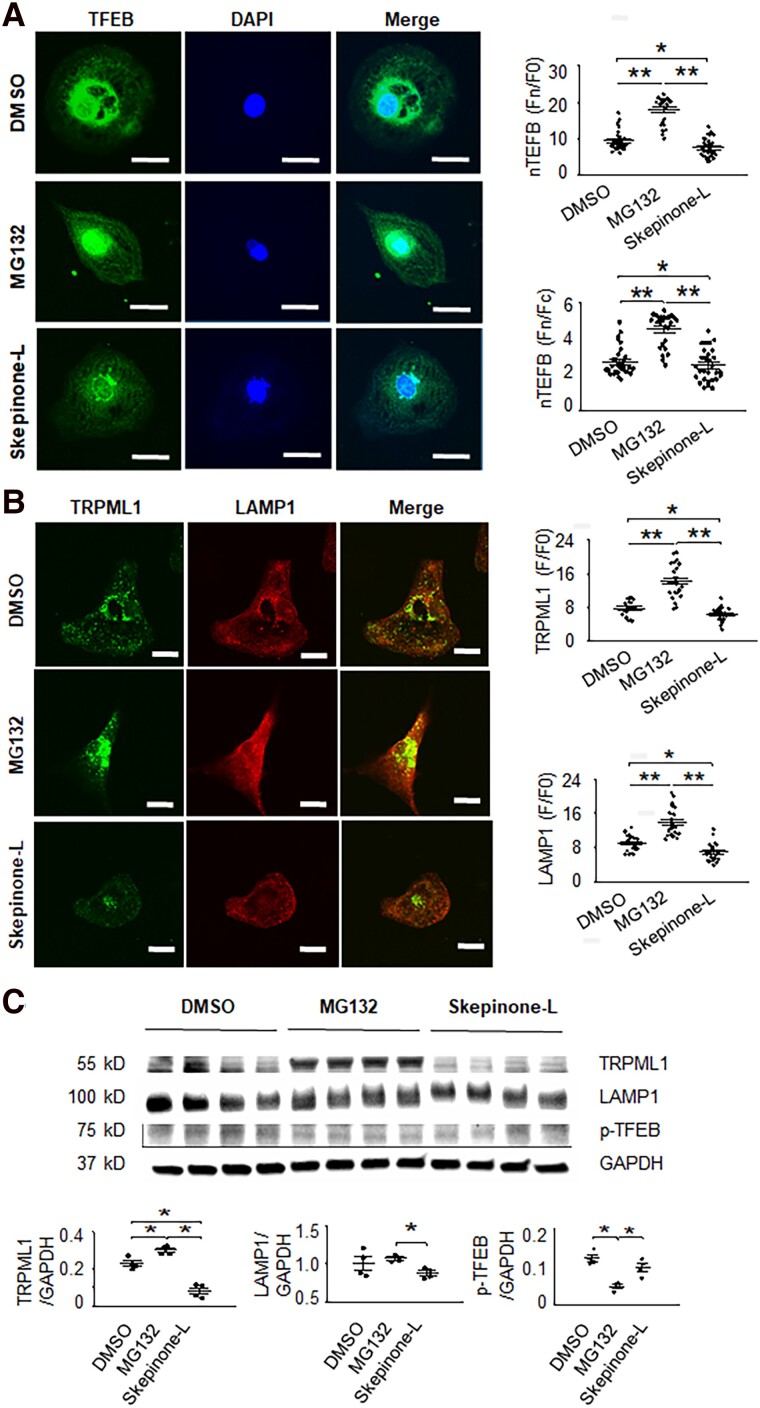
The change in expression of nuclear TFEB, TRPML1, and LAMP1 by MG132 and Skepinone-L in hiPSC-CMs. A) Proteasome inhibition by MG132 (15 μmol/L, 16 h) increased TFEB dephosphorylation and nuclear localization. On the other hand, proteasome activation by Skepinone-L (5 μmol/L, 16 h) decreased nuclear TFEB. bars: 20 μm. *n* = 31, 26, and 29 for DMSO vehicle, MG132, and Skepinone-L group, respectively. B) Proteasome inhibition by MG132 (15 μmol/L, 16 h) could increase TRPML1 and LAMP1 (lysosome markers). On the other hand, proteasome activation by Skepinone-L (5 μmol/L, 16 h) could decrease TRPML1 and LAMP1. bars: 20 μm. *n* = 20, 24, and 23 for DMSO, MG132, and Skepinone-L group, respectively. Data are represented as mean ± SEM. *P < 0.05 and **P < 0.01, compared with that between the two indicated groups by one-way ANOVA analysis. Bonferroni correction was used. C) Western blot data of TRPML1, LAMP1, and phosphorylated TFEB (p-TFEB, in cytosol) after CMs were treated with DMSO, MG132, and Skepinone-L, respectively.

**Fig. 5.**
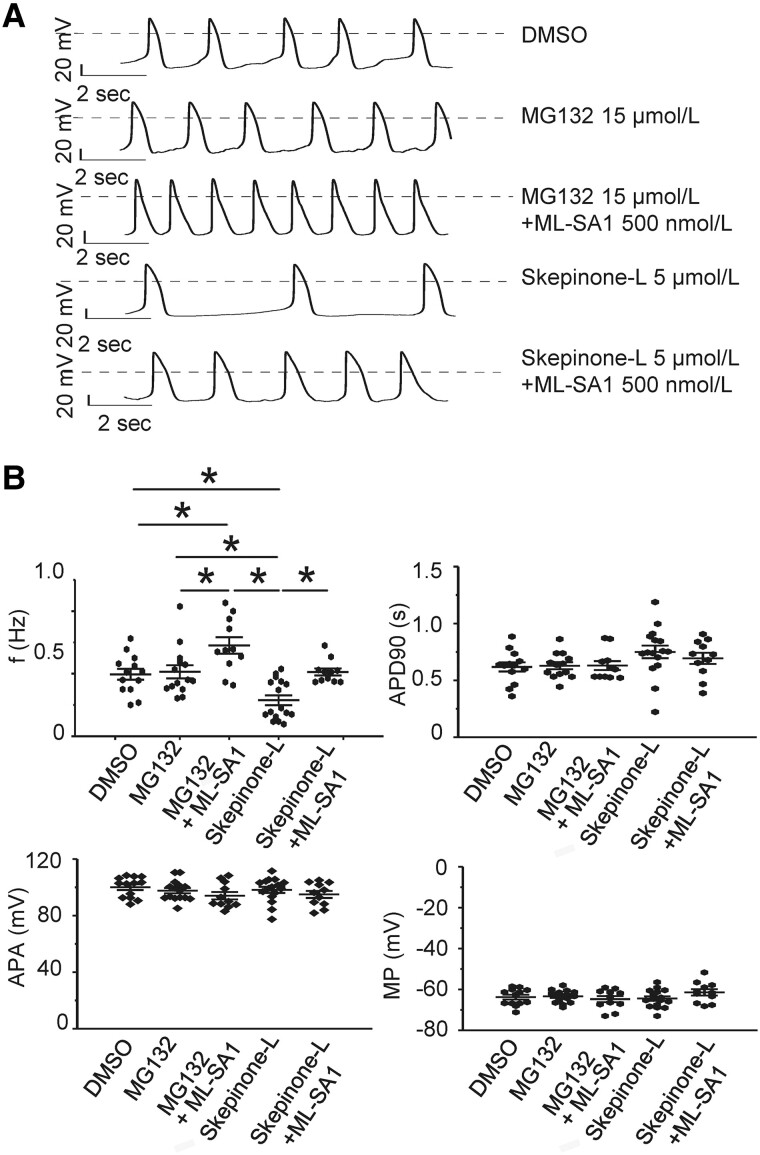
The automaticity alterations by MG132 and Skepinone-L in hiPSC-CMs. A) AP recordings showed that MG132 (a lysosome biogenesis inducer, 15 μmol/L, 16 h) did not increase significantly beating frequency, while Skepinone-L (a lysosome biogenesis inhibitor, 5 μmol/L, 16 h) could decrease the spontaneous beating rate. The dashed line indicates 0 mV. The TRPML1 agonist, ML-SA1 500 nmol/L, was able to accelerate the beating rate from the baseline after MG132 or Skepinone-L treatment. B) Summarized average beating frequency, AP duration at 90% repolarization (APD90), AP amplitude (APA), and maximum resting membrane potential (MP). Data are represented as mean ± SEM. *P < 0.05 and **P < 0.01, compared with that between two indicated groups by two-way ANOVA analysis. Bonferroni correction was used. *n* = 13, 13, and 15 for DMSO, MG132, and Skepinone-L group, respectively.

As shown previously, proteasome inhibition activates autophagy-lysosome pathways by enhancing TFEB dephosphorylation and nuclear translocation (24). As expected, the proteasome inhibitor MG132 (15 µmol/L, 16 h) could significantly enhance TFEB dephosphorylation and nuclear translocation (Fig. 4A and C), increasing lysosomes and TRPML1 protein expression (Figs. 4B and C and S1). On the other hand, proteasome activator Skepinone-L (5 µmol/L, 16 h) could significantly reduce TFEB dephosphorylation (nuclear translocation), TRPML1 levels, and lysosomal marker LAMP1 expression (Figs. 4 and S1). As expected, decreasing lysosomes reduced automaticity. Neither agent affected the AP, suggesting that the effect was not an unexpected change in membrane currents (Fig. 5). TRPML1 agonist ML-SA1 could increase automaticity in either case (Fig. 5A and B).

### Lysosomal Ca^2+^ release regulated automaticity in ischemic ventricular CMs and hiPSC 3D engineered heart tissues

**Fig. 6.**
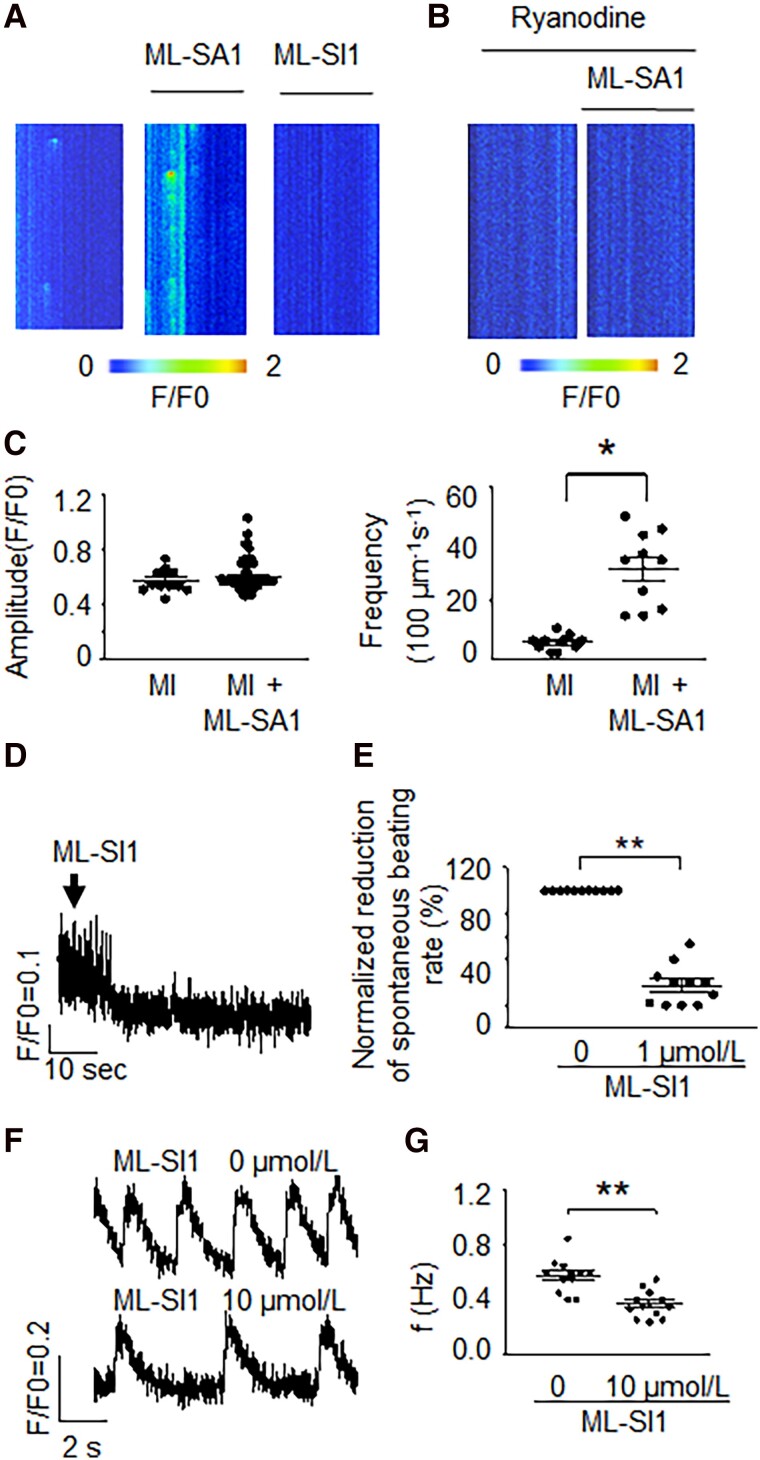
The inhibition of TRPML1 substantially decreased automaticity in ischemic ventricular CMs and 3D EHTs. A) Ca^2+^ sparks recorded before and after ML-SA1 (a TRPML1-specific agonist, 0.5 μmol/L) or ML-SI1 (a TRPML1-specific antagonist, 1 μmol/L, repeated in 10 cells) was applied in CMs isolated from MI mice. B) After SR Ca^2+^ release was blocked by a RyR2 blocker (ryanodine 10 μmol/L), 0.5 μmol/L ML-SA1 no longer induced Ca^2+^ sparks from CMs isolated from MI mice. Repeated in 10 cells. C) Summary of A. Data are represented as mean ± SEM. *P < 0.05, compared between two indicated groups by two-sample *t*-test. For amplitude comparison, *n* = 12 and 96 for MI control and MI + ML-SA1 groups, respectively. For frequency analysis, *n* = 10 (cell numbers) for each group. Each group was from three mice. D) The spontaneous beating recorded from MI ventricular CMs by Ca^2+^ transient recordings. E) Normalized reduction of spontaneous beating rate (%) after 1 μmol/L ML-SI1 was applied. Data are represented as mean ± SEM. **P < 0.01, compared with that in group before ML-SI1 was applied by Mann–Whitney *U* test. *n* = 11 for each group. F) The effect of TRPML1 antagonist (ML-SI1, 10 µmol/L, ∼2 min) on automaticity in hiPSC 3D EHTs. The two traces were from the same tissue construct. G) The average reduction of spontaneous beating rate (%) after 10 μmol/L ML-SI1 was applied. Data are represented as mean ± SEM. **P < 0.01, compared with that in group before ML-SI1 was applied by paired sample *t*-test. *n* = 12 for each group.

To show relevance to arrhythmogenesis, we performed experiments using CMs isolated from infarcted hearts, which are known to have increased arrhythmic risk. Ventricular CMs isolated from sham-operated animals showed no spontaneous beating. TRPML1 agonist ML-SA1 could increase Ca^2+^ spark frequency in myocardial infarction (MI) CMs (Fig. 6A and C), which could be eliminated by SR RyR2 inhibition (Fig. 6B). Moreover, the TRPML1-specific antagonist, ML-SI1 (1 µmol/L), abolished Ca^2+^ sparks in MI CMs (Fig. 6A). Control ventricular CMs are usually quiescent unless injured (29). Acutely isolated adult mouse ventricular CMs from infracted hearts showed abnormal automaticity. Consistent with the hiPSC-CMs, data showed that the TRPML1-specific antagonist, ML-SI1 (1 µmol/L), could decrease substantially this abnormal automaticity (Fig. 6D and E), suggesting that the findings were relevant across species and to ventricular arrhythmic risk. Control hearts had less TRPML1 expression and no spontaneous beating and the TRPML1 activator could not induce automaticity in control (sham) CMs (Fig. S2). Furthermore, the effect of TRPML1 inhibition in 3D engineered heart tissue (EHT) by ML-SI1 (10 µmol/L) was similar to that in isolated CMs, suggesting relevance of the effect to intact tissue (Fig. 6F and G).

### TRPML1 protein expression increased in ventricular tissues from heart failure patients with ventricular tachycardia

**Fig. 7.**
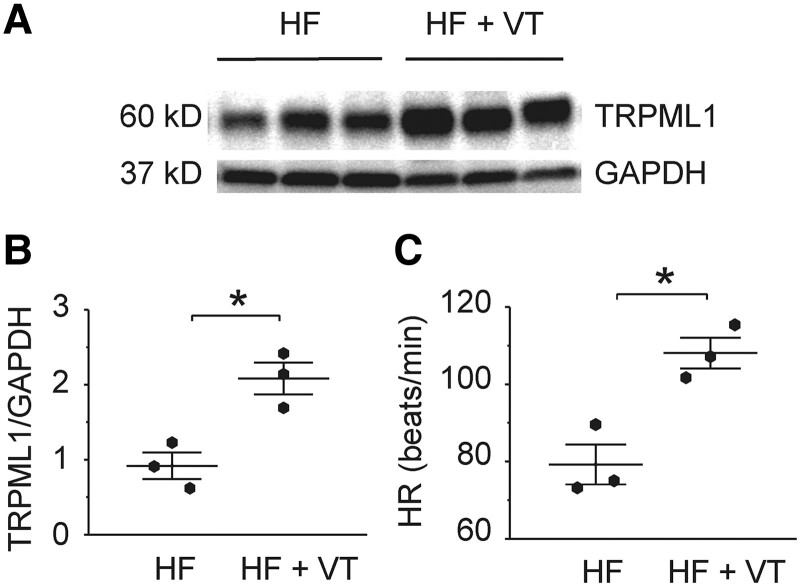
The ventricular tissue TRPML1 protein level and the heart rate of HF patients with and without VT. A) and B) The Western blot of TRPML1 in ventricular tissues from HF patients with and without VT. C) Heart rate (HR) of HF patients with and without VT. QRS was less than 120 ms, and the EF was between 10 and 20% for all the HF patients. Data are represented as mean ± SEM. *P < 0.05, compared with those in the patients without VT by the Mann–Whitney test. *n* = 3 for each group.

To suggest human relevance of our findings, ventricular TRPML1 was measured in cardiomyopathic heart failure (HF) patients with and without reported ventricular arrhythmias. The protein level of TRPML1 in HF patients with ventricular tachycardia (VT) was increased when compared with those subjects without VT (Fig. 7, *P* < 0.05).

## Discussion

**Fig. 8.**
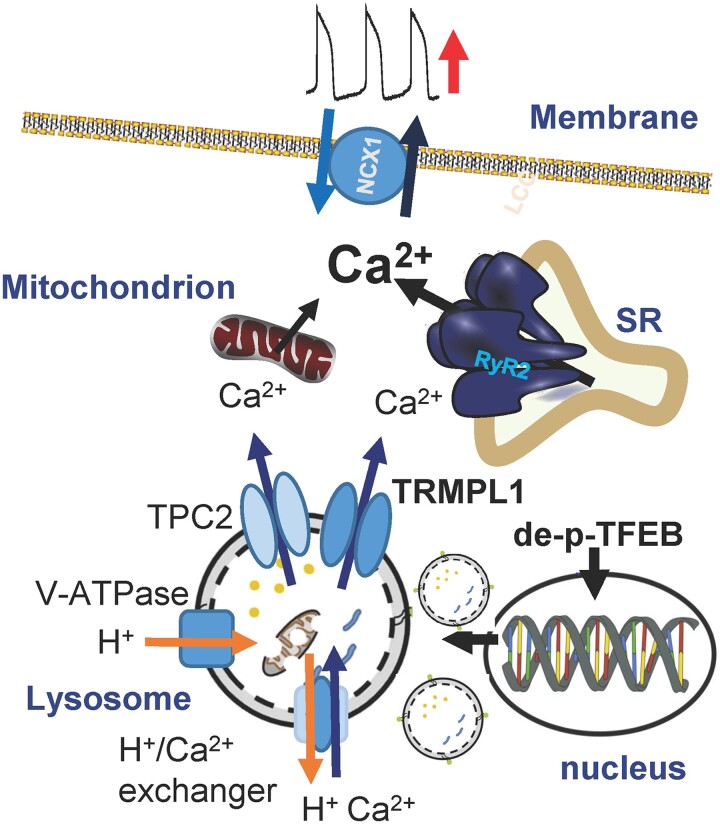
Lysosome Ca^2+^-related ion channels and transporters contribute to ventricular automaticity. Up-regulation of lysosomal Ca^2+^ release increased abnormal automaticity, while reduced lysosomal Ca^2+^ release reduced automaticity. It is unclear if lysosomal Ca^2+^ release mediates its effects directly or via SR or mitochondrial Ca^2+^ release. NCX1, type 1 Na^+^/Ca^2+^ exchanger; SR, sarcoplasmic reticulum; RyR2, type 2 ryanodine receptor; TRPML1, transient receptor potential mucolipin channel (TRPML1); TPC2, type 2 two-pore channel; de-p-TFEB, dephosphorylated transcription factor EB; V-ATPase, V-type H^+^-ATPase.

Lysosomes are critical to autophagy and mitophagy. As part of this function, they maintain an increased intraluminal Ca^2+^ concentration (∼500 µmol/L) (22). In the heart, lysosome Ca^2+^ release is mediated by TPC2 and TRPML1 channels (21). The Ca^2+^-permeable, nonselective cation channel known as TRPML1 is found exclusively on the membranes of late-stage endosomes and lysosomes, and it is not present or active on the plasma membrane (18, 23). TRPML1 activity is regulated by lysosomal luminal Ca^2+^, luminal acidity, and ROS (28, 30). TPC2 is a nicotinic acid adenine dinucleotide phosphate (NAADP)–sensitive Ca^2+^ release channel (31). TRPML1 is considered to be the principal Ca^2+^ release channel in the lysosome (26). The energy necessary for the entry of Ca^2+^ into lysosomes through the H^+^/Ca^2+^ exchange mechanism is supplied by the V-ATPase's activity (21). Therefore, inhibition of the V-ATPase can decrease lysosomal Ca^2+^ uptake. The lysosome Ca^2+^ cycling pathway is described in Fig. 8.

Endo-lysosomal TRPML1 channels can trigger global SR Ca^2+^ release and Ca^2+^ influx (22). TRPML1 can trigger Ca^2+^ sparks (20). In addition, the contact between mitochondria and lysosomes is responsible for the regulation of mitochondrial Ca^2+^ dynamics through lysosomal TRPML1 (32). Ca^2+^ oscillations such as mitochondrial Ca^2+^ cycling, SR Ca^2+^ release, and membrane Ca^2+^ influx have effects on automaticity (11). Therefore, it is plausible that lysosome Ca^2+^ handling may also affect automaticity. Our pharmacological and genetic manipulations of lysosome Ca^2+^ homeostasis through modulation of TRPML1, TPC2, or V-ATPase support this hypothesis. Consistent with Ca^2+^ cycling being central to automaticity, increasing the TRPML1 channel activity could enhance automaticity, and decreasing the lysosome Ca^2+^ release channel or blocking of the lysosome Ca^2+^ uptake pathway could reduce spontaneous beating of ventricular CMs and hiPSC 3D EHTs. Paradoxically, increased TRPML1 mRNA (Fig. 3A) or protein (Figs. 3A and 4B) did not increase ventricular automaticity (Figs. 3B and C and 5), while increased TRPML1 channel activity increased abnormal automaticity (Fig. 2A, 3B, and 5). That down-regulation and inhibition of TRPML1 reduced automaticity, but overexpression of the channel alone did not increase automaticity despite the TRPML1 agonist activity influencing automaticity suggests that channel up-regulation alone is insufficient to affect automaticity and that there are other factors determining channel activity that are important for influencing automaticity.

Inhibiting proteasomal degradation of TFEB stimulates lysosomal production associated with nuclear translocation of dephosphorylated TFEB (24). Dephosphorylation of TFEB is known to regulate its nuclear localization (28). Once localized in the nucleus, dephosphorylated TFEB acts as a transcription factor to increase lysosomal production, including TRPML1 transcription (Fig. 8). Increased lysosomal production is thought to enhance subsequent autophagy and mitophagy (28). Altering TFEB has similar effects to the more direct modulation of lysosomal Ca^2+^ release, suggesting increased lysosomal activity could also enhance automaticity. Since lysosomal activity is increased in many cardiomyopathic conditions, this observation suggests that abnormal automaticity may be enhanced by increased lysosomal function. HF patients had increased TRPML1 expression accompanied with VT events. This data supported the concept that lysosomal Ca^2+^ cycling may increase cardiac arrhythmic risk by enhancing abnormal automaticity. This idea was also supported by reduced VT inducibility of isolated hearts exposed to TRPML1 inhibition (Fig. 7).

The electrophysiology of ventricular-like hiPSC-CMs is similar to but not identical with native ventricular cells. For example, hiPSC-CMs have down-regulated I_K1_ and up-regulated I_f_ currents if compared with acutely isolated ventricular cells (25). Nevertheless, these cells have been used widely as a platform to model arrhythmogenic diseases (33), and many characteristics of automaticity are similar to mature CMs. For example, both SR and mitochondrial Ca^2+^ handling are involving in the automaticity in these two types of CMs (11). The experiments with acutely isolated ventricular myocytes suggest that the role of lysosomes in automaticity is conserved and can be modeled using hiPSC-CMs. It is possible that a bad isolated cell preparation can cause injury and ventricular automaticity. Nevertheless, myocytes isolated from sham hearts did not show automaticity (Fig. S2), while those from MI hearts did (Fig. 6D and E). Moreover, TRPML1 antagonism was able to suppress almost all automaticity, suggesting a specific effect rather than generalized cell injury.

It seems clear from the literature that cell Ca^2+^ handling is an important determinant of automaticity. Cell Ca^2+^ cycling is complicated with multiple sites of potential oscillators including the membrane, the SR, and the mitochondria. In this manuscript, we show for the first time that lysosomal Ca^2+^ can play a role in ventricular CM and hiPSC 3D EHT automaticity. It is unclear if lysosomal Ca^2+^ release directly affects local membrane currents or acts to modulate SR or mitochondria Ca^2+^ to have its effect. The rapidity of the alteration in automaticity in the pharmacological experiments suggests that the lysosomal effect can be achieved without altering lysosomal location or other functions than Ca^2+^ handling or relation to other organelles or the membrane.

In summary, lysosomal Ca^2+^ release can enhance automaticity of ventricular CMs and is associated with arrhythmia in isolated hearts, heart constructs, and human HF. Enhanced lysosomal activity in cardiomyopathic states may help explain abnormal automaticity and increased arrhythmic risk in cardiomyopathy and may represent a new target for antiarrhythmic therapy.

## Materials and methods

### Culture and differentiation of hiPSC-CMs

The hiPSC-derived CMs (iCell Cardiomyocytes^2^, 01434) were purchased from FUJIFILM Cellular Dynamics, Inc. (Santa Ana, CA, USA) and differentiated for 30 days. Cell plating and maintenance were carried out according to the protocol in the manufacturer's instructions. To reduce the variability of the beating rate, CMs with the lowest quartile beating rates in the same culture conditions were selected for comparison, and the experiments were repeated in at least three different differentiation CM batches. Ventricular hiPSC-derived CMs were selected by action potential (AP) characteristics. Ventricular-like APs had maximum diastolic potentials close to those of human CMs (25). When using these cells, the observers were blinded to the treatment group.

### Ischemic HF model

The animal protocols for this study were in accordance with the guidelines of the Animal Care and Use Committee of the University of Minnesota and the Guide for the Care and Use of Laboratory Animals published by the National Institutes of Health. Mice of either sex were randomly selected for the treatment or sham groups. The observers were blinded to the treatment group. FVB/NJ mice were brought from the Jackson Laboratories (Bar Harbor, ME, USA). MI was induced in 11- or 12-week-old mice (25–35 g, either sex) by coronary artery occlusion (34). Mice were anaesthetized using inhaled isoflurane (3% for induction and 1.5% for maintenance) and ventilated after tracheal intubation by positive pressure respirator (Harvard Apparatus, Holliston, MA, USA). A left thoracotomy was performed just lateral to the sternum to expose the heart. The pericardium was removed, and an 8-0 monofilament suture was looped around the proximal left anterior descending coronary artery. The suture was either knotted to induce infarction or removed in the sham surgery. The intercostal muscles, pectoralis muscle, and skin were sutured, and the animals were allowed to recover on a heating pad at 37°C. The mice were treated with slow-release buprenorphine and monitored postoperatively for 3 days. The average infarction area of nine MI hearts was ∼33%. Three weeks after MI, hearts were used for CM isolation.

### CM isolation

Ventricular CMs were isolated as we described before (35, 36). CMs were placed at room temperature for 1 h prior to being used for patch clamp recording and Ca^2+^ transient measurements.

### hiPSC-CMs intracellular Ca^2+^ transient measurements

Fluo-4 AM (3 µmol/L, ThermoFisher Scientific, Minneapolis, MN, USA) loaded for 20 min followed with 20 min of de-esterification was employed to measure cytoplasmic Ca^2+^ transients in hiPSC-CMs (11). Ca^2+^ transients were recorded before and after chemical compounds were applied for about 2 min at room temperature.

For lysosomal TRPML1 Ca^2+^ release measurements, Fluo-4 was loaded as above. For imaging, the time-lapse mode with a frame interval of 3 s was used. In TRPML1-CFP overexpressing hiPSC-CMs, cells with CFP fluorescence were chosen. Their Fluo-4 fluorescence images and traces were recorded from these cells. To measure the small lysosomal Ca^2+^ release, we focused on only the Fluo-4 fluorescence.

### Electrophysiological recordings

AP was recorded as we described before (37). A total of 0.02 mmol/L EGTA and 0.05 mmol/L CaCl_2_ was added to pipette solution to keep free [Ca^2+^]_i_ ≈ 50 nmol/L. Junction potentials were compensated when membrane potentials were calculated. APs were recorded before and after chemical compounds were applied for about 2 min at room temperature.

### Silencing TRPML1 and overexpression of TRPML1-CFP

To silence TRPML1, specific TRPML1 siRNA and scrambled siRNA were purchased from Santa Cruz Biotechnology, Inc. (Dallas, TX, USA). TRPML1-CFP was a gift from Craig Montell (Addgene plasmid, Catalog # 18827, Watertown, MA, USA). Cells were transfected by TransIT-siQUEST Transfection Reagent (Mirus Bio, Madison, WI, USA) for 72 h with 50 nM siRNA and by FuGene6 transfection reagent (Promega, Madison, WI, USA) with 1 μg DNA. TRPML1 mRNA was measured after 72 h. qPCR was performed using PowerUp SYBR Green Master Mix (ThermoFisher Scientific). The primer set is as follows: hMCOLN1_F-CGG ACT GCT ATA CCT TCA GCG T; hMCOLN1_R-GGT GCT TAC ACT CCT GGA TGT G, and hGAPDH_F-GAA GGT GAA GGT CGG AGT CAA C; hGAPDH_R-CAG AGT TAA AAG CAG CCC TGG T. Primers were purchased from Integrated DNA Technologies (Newark, NJ, USA).

### Immunofluorescence staining

CMs were fixed with 4% formaldehyde for 15 min at room temperature. Then, they were rinsed three times in 1XPBS for 5 min each followed with blocking for 60 min. After aspirating the blocking solution, primary antibodies were incubated overnight at 4°C and rinsed three times in 1XPBS for 5 min each. Primary antibodies were TRPML1 (ACC-081, Alomone, Jerusalem, Israel. 1:200), LAMP1 (Cell Signaling Technology, Danvers, MA, USA. 1:200), and TFEB (MyBioSource, Inc., San Diego, CA, USA. 1:150). CMs were incubated in fluorochrome-conjugated secondary antibody diluted in Antibody Dilution Buffer (1:500) for 1–2 h and rinsed three times in 1XPBS for 5 min each while being protected from light. Images were acquired by confocal microscope (Olympus FluoView FV3000. Tokyo, Japan) with FluoView FV31S-SW software.

### Western blotting analysis

Western blotting and left ventricular cardiac tissues samples preparation were performed as we described before (37). The anti-TRPML1 antibody (1:500, Abcam, Cambridge, MA, USA) or lysosomal marker LAMP1 antibody (C54H11, 1:1,000, Cell Signaling Technology, Inc., Danvers, MA, USA) or p-TFEB S211 antibody (E9S8N, 1:500, Cell Signaling Technology, Inc.) was used as primary antibody. HF heart tissues, ejection fractions (EF), and ECGs were obtained from the Lillehei Heart Institute (University of Minnesota) tissue bank.

### Ca^2+^ sparks images recorded from left ventricular CMs

The method was described previously (38). Ca^2+^ sparks were recorded from the same batch of CMs before and after chemical compounds were applied for about 2 min at room temperature.

### Generation of EHTs and their Ca^2+^ transient recording

The EHT negative mold was printed on a Stratasys J750 PolyJet 3D Printer using digital ABS plastic. The ABS mold was thoroughly cleaned, oxygen plasma treated for 10 min (PDC-32G, Harrick Plasma), and treated with silane vapors for 24 h in a desiccator vacuum. Sylgard 184 PDMS precursor was mixed at a 1:10 mass ratio with PDMS curing agent and cast onto the ABS negative mold. The curing PDMS was degassed for 1 h at room temperature and cured overnight at 50°C. The resultant PDMS mold contained 12 rectangular EHT culture wells, and each EHT well contained 2 posts. The PDMS mold was further cured at 50°C for 1 week. The mold was then cut into six two-mold squares that were sonicated in 70% ethanol for 30 min and autoclaved at 121°C for 25 min.

The sterile two EHT well PDMS squares were transferred into a sterile petri dish and oxygen plasma treated for 2 min. The molds were then transferred to a 12-well plate and treated with 0.5 *w*/*v*% Pluronic F-127 for 30 min at room temperature. The hiPSCs were differentiated into CMs using small molecule WNT modulation. Briefly, the hiPSC (the cell line hciPSC-MHC-CCND2 was kindly provided by Dr. Jay Zhang, the University of Alabama at Birmingham, AL, USA) were plated at a cell number of 0.5 × 10^6^ per well of a Matrigel (Corning)–coated 12-well plate. The cells were grown in mTesR1 (Stemcell) for 2–3 days until they reached 100% confluency. On day 0 of differentiation, the hiPSC were treated with 6.5 μM CHIR99021 (Sigma Aldrich) for 48 h following treatment with 7.5 μM IWP2 (Tocris) for 48 h in RPMI supplemented with 1X B27 minus insulin supplement (RPMI/B27/−ins). After IWP2 treatment, on day 4 of differentiation, the media was replaced with fresh RPMI/B27/−ins. Starting on day 6 until day 13, the media was replaced every 48 h with RPMI supplemented with 1X B27 supplement (RPMI/B27/+ins). On day 13, the hiPSC-CMs, along with other contaminating cells, were dissociated with 0.25% Trypsin and split at a 1:2 ratio onto new Matrigel-coated plates. The cells were left to recover for 48 h, and then, they were lactate purified using DMEM (−)glucose (Gibco) that was supplemented with 4 mM sodium l-lactate (Sigma Aldrich) for 4 days. The purified hiPSC-CMs were recovered in RPMI/B27/−ins until day 22 after differentiation initiation. Neonatal human dermal fibroblasts (nHDF, Lonza CC-2509, kindly provided by Dr. Robert Tranquillo, the University of Minnesota, MN, USA) were expanded and maintained in DMEM-high glucose with 20% EmbryoMAX FBS (Millipore). At day 22 of hiPSC-CM differentiation, the purified hiPSC-CMs were dissociated in 0.25% Trypsin-EDTA and combined with nhDF that were singularized in Accutase (Millipore). The hiPSC-CMs and nHDF were combined at a 3:1 ratio and a density of 10 × 10^6^ cells/mL in fibrin gel mixture containing 20 mg/mL fibrinogen from human plasma, RPMI/B27/+ins with 20 μM Rock Inhibitor (VWR), and 100 U/mL thrombin from human plasma (Sigma) at a 6:3:1 ratio. Each EHT culture well was seeded with 100 μL of fibrin gel cell suspension, which immediately formed a gel at room temperature. The EHTs were then cultured in RPMI/B27/+ins with 10 μM Rock Inhibitor and 20 μg/mL aprotinin for 24 h. Then, the EHTs were given fresh RPMI/B27/+ins with 20 μg/mL aprotinin every 2 days until assessment on day 16.

Indo-1 AM (ThermoFisher Scientific, Minneapolis, MN, USA, 3 μmol/L) were used to record the Ca^2+^ transients in EHTs as we used before (11). A slice anchor (SHD-26H/15, WI: 64-0251, Warner Instruments, LLC, Holliston, MA, USA) was used to restrict tissue movement. Three batches of EHTs were used for Ca^2+^ transient measurements.

### Statistics

Data are shown as the mean ± SEM. As noted in the text, the Mann–Whitney test or paired or two-sample *t*-test was employed for statistical analysis. Bonferroni correction was used in one-way and two-way ANOVA tests for multiple comparisons. A value of *P* < 0.05 was considered statistically significant. OriginPro 2017 SR2 (version b9.4.2.380, OriginLab, Northampton, MA, USA) was used for statistical analysis.

## Supplementary Material

pgad174_Supplementary_Data

## Data Availability

All data are available in the main text.
