# Supplementary material for: Lysosomal Ca2+ flux modulates automaticity in ventricular cardiomyocytes and correlates with arrhythmic risk
Source: PNAS Nexus. 2023 May 25;2(6):pgad174. doi: 10.1093/pnasnexus/pgad174 (PMC10255768; doi:10.1093/pnasnexus/pgad174)
Supplement: pgad174_Supplementary_Data [file pgad174_supplementary_data.docx]

**
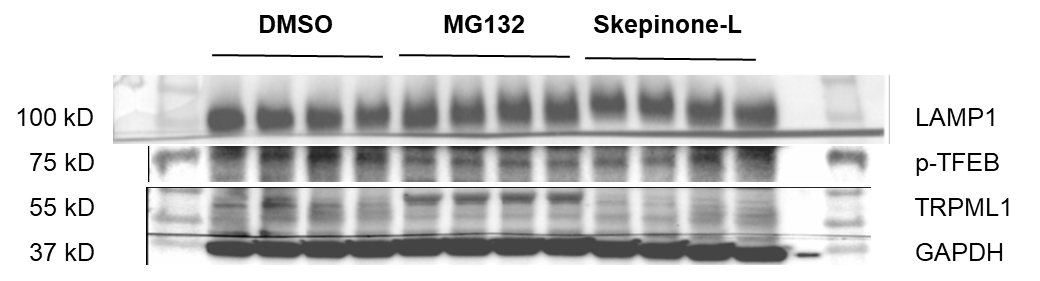
**

**Figure S1. Uncropped Western blot data of TRPML1, LAMP1, and phosphorylated TFEB (p-TFEB, in cytosol) after CMs were treated with DMSO, MG132 and Skepinone-L, respectively.**

**
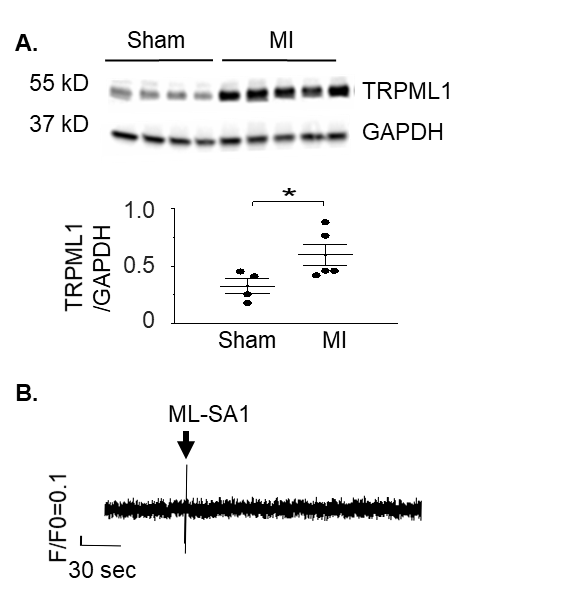
**

**Figure S2. Cardiomyocytes isolated from sham mice had less TRPML1 expression, and no spontaneous beating could be evoked by TRPML1 agonist ML-SA1.**

**A.** Western blot data of TRPML1 from sham and MI heart tissues. Data represented as mean ± SEM. **p*<0.05, compared with that in sham group by Mann-Whitney test. n=4 and 5 for sham and MI group respectively. **B.** TRPML1 activator, 500 nmol/L ML-SA1, could not induce automaticity in control (sham) cardiomyocytes. Repeated in 12 cells from three mice.
